# Carboxylato-Pillar[6]arene-Based Fluorescent Indicator Displacement Assays for Caffeine Sensing

**DOI:** 10.3389/fchem.2021.816069

**Published:** 2021-12-21

**Authors:** Qunpeng Duan, Yibo Xing, Kainan Guo

**Affiliations:** School of Chemical and Printing-dyeing Engineering, Henan University of Engineering, Zhengzhou, China

**Keywords:** pillararene, host-guest complex, fluorescent, indicator displacement assay, caffeine sensing

## Abstract

In the present work, we have developed a new indicator displacement system based on pillararene for anionic water-soluble carboxylato pillar [6] arene (WP6) and aromatic fluorescent dye safranine T (ST). A large fluorescence enhancement and colour change of ST were observed after complexation with electron-rich cavity in WP6 because of host-guest twisted intramolecular charge-transfer interactions. The constructed pillararene-indicator displacement system can be applied for caffeine selective detection in water.

## Introduction

Fluorescent indicator displacement assays (F-IDAs) are typically used to convert synthetic receptors into optical sensors in supramolecular chemistry. In F-IDAs, the competitive binding principle is used: after binding a fluorescent indicator to the receptor, when a competing analyte is introduced into the indicator–receptor pair, the indicator is discharged from the receptor to induce a fluorescence change (Wiskur, et al., 2001;Nguyen and Anslyn, 2006). Macrocyclic hosts typically provide ideal receptors for use because of their particular composition and excellent functions. The macrocyclic hosts, such as cyclodextrins (Crini, 2014; Pal, et al., 2015), calixarenes (Koh, et al., 1996; Hennig, et al., 2007; Guo and Liu, 2014; Zheng, et al., 2018), cucurbiturils (Florea and Nau, 2011; Praetorius, et al., 2008; Barrow, et al., 2015; Sonzini, et al., 2017) and pillararenes (Wang P, et al., 2014; Bojtár, et al., 2015; Bojtár, et al., 2016; Hua, et al., 2016; Bojtár, et al., 2017; Hua, et al., 2018; Xiao, et al., 2018, Xiao, et al., 2019a; Xiao, et al., 2019b), combined with various dyes have been applied as receptors in F-IDAs for specific and selective sensing in drugs, biomolecules, or other organic compounds.

**SCHEME 1:**
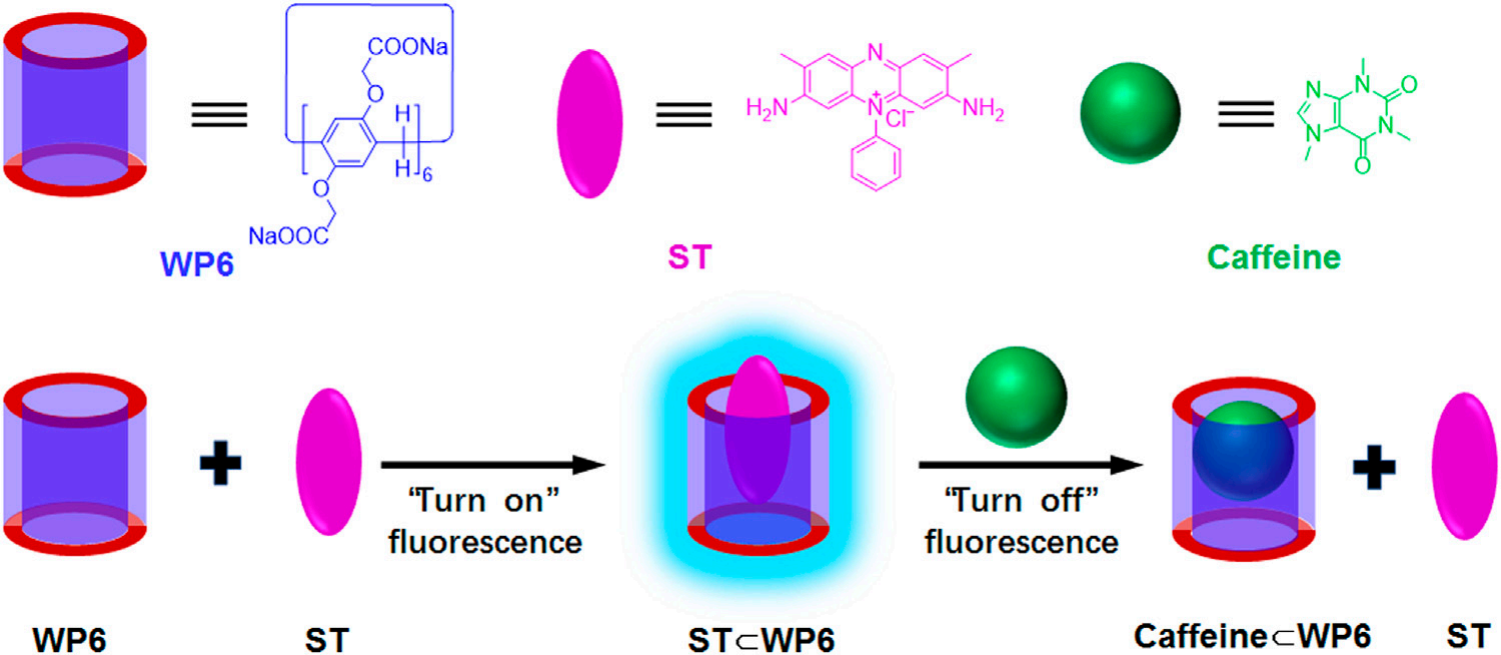
Chemical structures and cartoon presentations of WP6, ST and caffeine and illustration of the turn-off fluorescence detection of caffeine through indicator displacement process.

This study established an FID assay with a water-soluble pillararene for caffeine detection. Caffeine is the most widely consumed psychostimulant drug worldwide. Appropriate caffeine intake may enhance alertness, attention, and nerve cell activity and decrease the possibility of type 2 diabetes. However, excessive intake of caffeine may possibly cause a headache, high blood pressure, irregular small muscle movement, and allergy, especially in teenagers and pregnant women (Nehlig, et al., 1992; Rapuri, et al., 2001; Smith, 2002; Lovallo et al., 2005). Caffeine detection can be realised with costly and complex methods, such as HPLC-MS and immunoassay (Wu, et al., 2000; Oberleitner, et al., 2014). Therefore, caffeine detection remains inconvenient for public usage. Thus, we realised novel host–guest recognition between water-soluble pillararene (WP6) and safranine T (ST) and revealed the operation of this host–guest recognition motif as an FID assay in caffeine detection (Scheme 1). The assay seems selective for theophylline and theobromine.

## Materials and Methods

The reagents used were marketable and applied directly without further purification. WP6 (Yu et al., 2012) was synthesized by following the known procedures. Nuclear magnetic resonance (NMR) spectra were obtained using the Bruker Avance III HD 400 spectrometer with the deuterated solvent as the lock and the residual solvent as the internal reference. Fluorescence spectra were obtained by using the Agilent Cary Eclipse fluorescence spectrophotometer. To prevent the dilution effect during titration, WP6 stock solutions were produced using the same ST solution. The measurement was repeated three times for each experiment. Displacement assay for theophylline and theobromine was performed at pH 7.2 with WP6 at varying concentrations of theophylline and theobromine, respectively. All the experiments were conducted at room temperature (298 K).

## Results and Discussion

### Complexation of ST With WP6

**FIGURE 1:**
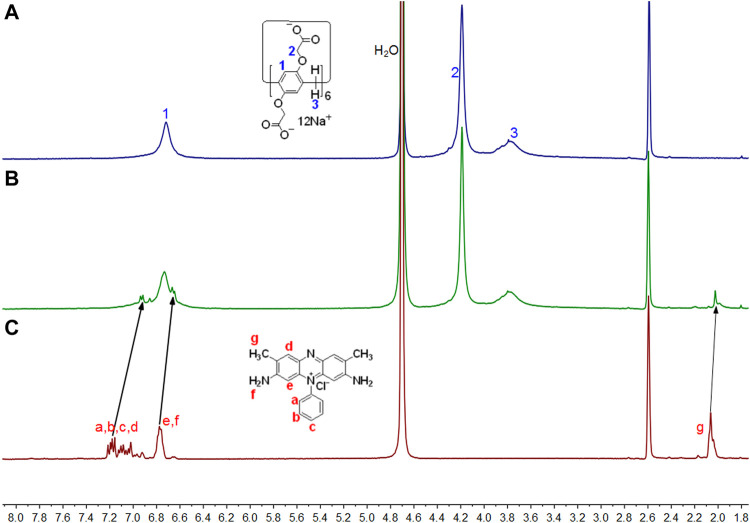
Partial ^1^H NMR spectra (400 MHz, D_2_O:DMSO-*d*_6_ = 1:1, 298 K) for **(A)** 3 mM WP6, **(B)** 3 mM WP6 and 10 mM ST, **(C)** 10 mM ST.

To study the host–guest complexation between WP6 and ST, ^1^H NMR spectroscopy was first performed. Given that the complex solubility of neat D_2_O did not occur at the mM scale, DMSO-*d*_6_ cosolvent was supplemented. According to Figure 1, ST aromatic protons in the complex shifted upfield to varying degrees. This result revealed that ST was encapsulated by WP6 cavity and protons on ST were shielded by the electron-rich cyclic structure when the inclusion complex formed (Wang Y. et al., 2014). The characteristic signal broadening of the protons on ST was observed because of the shielding effects of the aromatic host (Li et al., 2010). Furthermore, protons on WP6 revealed minor chemical shifts resulting from host–guest interactions between WP6 and ST.

**FIGURE 2:**
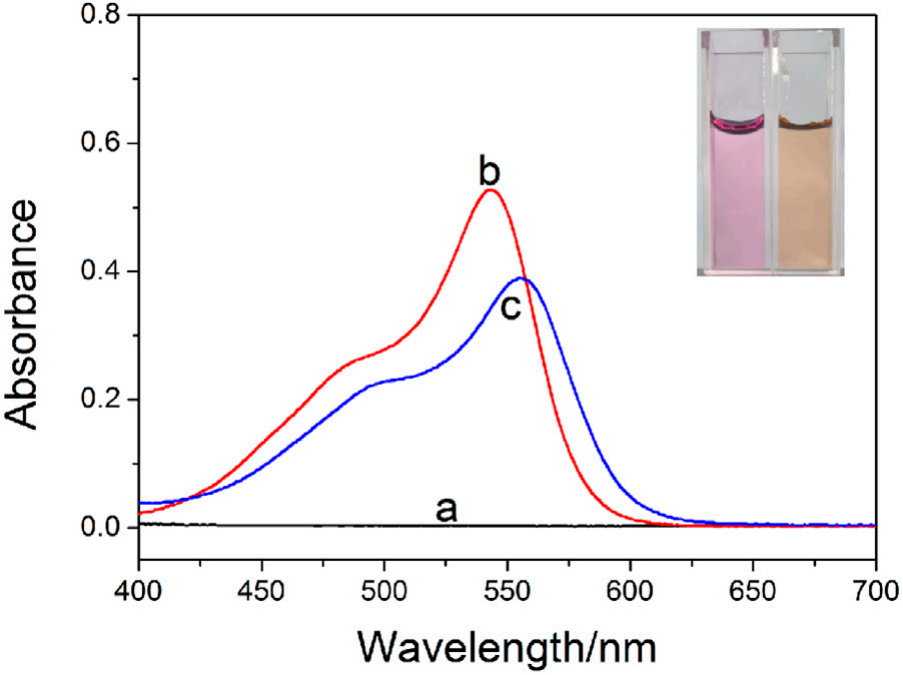
UV-vis spectra for **(A)** WP6, **(B)** ST, and **(C)** ST in the presence of 50 equiv. of WP6 (1 × 10^−3^ M) in PBS (pH = 7.2). The inserted photo displays the colour changes related to ST with the addition of WP6.

**FIGURE 3:**
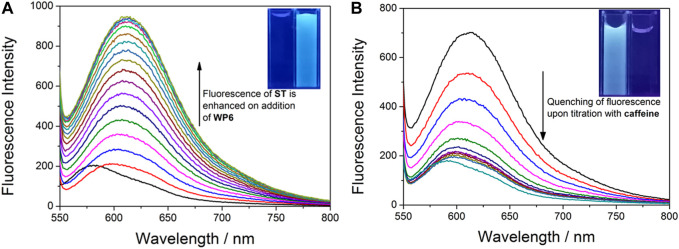
**(A)** Changes of the fluorescence intensity in ST (0.02 mM) upon the titration of WP6 (0–25 equiv.) in PBS (λ_ex_ = 523 nm, λ_em_ = 584 nm, pH = 7.2). The inserted photo exhibits an enhancement in fluorescence in water under excitation at 365 nm via the UV lamp at 298 K. **(B)** Fluorescence titration for the competitive displacement of ST (0.02 mM) from WP6 (0.3 mM) using caffeine (0–150 equiv.) in PBS at pH 7.2 (λ_ex_ = 523 nm, λ_em_ = 584 nm). The inserted photo exhibits the corresponding fluorescence quenching in water under excitation at 365 nm via the UV lamp at 298 K.

The formation of host–guest complex between WP6 and ST was further confirmed through UV-vis absorption spectroscopy. (Figure 2). A broad absorption band above 555 nm, corresponding to the charge-transfer interaction between electron-rich WP6 and electron-deficient ST, was observed. Furthermore, after adding WP6 to ST, a red shift appeared, which indicated that a representative charge-transfer complex was formed (Wang Y et al., 2014). The fluorescence titration of ST with WP6 was performed under ambient temperature in water. According to. Figure 3A, an enhancement in fluorescence and a red shift in the emission spectra were observed with the progressive supplement of WP6, which indicated that a strong supramolecular complex was formed. These changes may arise from the formation of twisted intramolecular charge transfer (TICT) state when ST occupied the WP6 cavity in the aqueous buffer. Under the TICT state, the phenyl or phenazinyl group is assumed to rotate around bonds that connect them to the central single bond. The twisting movement is subjected to restriction of the encapsulated **ST** guest, leading to enhanced fluorescence (Grabowski et al., 2003; Bojtár, et al., 2015).

The association constant (*K*_a_) and the optical spectroscopic data of ST and corresponding WP6 complex are listed in Table 1, which presents a comparison with the data of ST complexes with β-cyclodextrin, ST⊂β-CD (Zhang et al., 2005), disulphide bridged β–cyclodextrin, ST⊂SS-β-CD (Yang et al., 2017), and γ-cyclodextrin, ST⊂γ-CD (Wang et al., 2012). An association constant of *K*_a_ = (1.50 ± 0.06) × 10^4^ M^−1^ was obtained using a nonlinear fitting to the fluorescence spectra, measured by titration experiments. The stoichiometry of 1:1 for the complexes was tested with the molar ratio approach, based on the fluorescence data related to WP6-ST mixtures.

**TABLE 1:** Association constants (*K*_a_) and optical spectroscopic data for the complexes of **ST** with **WP6** and other macrocycles.

	*K*_a_	λabs (nm)	λem (nm)	References
ST	—	523	577	This work
ST⊂β-CD	160 M^−1^	525	573	Zhang et al., 2005
ST⊂SS-β-CD	4.7 × 10^3^ M^−1^	525	576	Yang et al., 2017
ST⊂γ-CD	7.41 × 10^3^ M^−1^	411	579	Wang et al., 2012
ST⊂WP6	(1.50 ± 0.06) × 10^4^ M^−1^	555	584	This work

The optimal association constant revealed system applicability to FID. The spectroscopic data for complex ST⊂WP6 were similar to the spectroscopic data for the complex of the dye containing SS-β-CD, ST⊂β-CD (Yang et al., 2017), which proved the similarity of polarity of these two macrocycles. However, because of the weak interactions, the association constant with the uncharged cyclodextrin was lowered by an order of magnitude.

### Complexation of Caffeine, Theophylline, and Theobromine With WP6

Next, for evaluating analyte complexation, ^1^H NMR spectra were obtained for caffeine, theophylline, and theobromine. Supplementary Figure S1 reveals that all the proton signals of caffeine shifted upfield at various extents, which indicated that caffeine was threaded into the host cavity. Furthermore, according to the 2D NOESY spectrum (Supplementary Figure S4), NOE correlation signals were obtained between protons H_a-d_ of caffeine and proton H_1_ on WP6, verifying WP6’s assignment for the caffeine threaded structure. Signals from the NMR spectra for theophylline and theobromine exhibited similar changes after adding WP6 (Supplementary Figures S2, S3).

Next, fluorescence titrations were performed at 298 K in PBS at pH 7.2 for estimating the binding behaviours of WP6 with caffeine, theophylline, and theobromine in a quantitative manner. Job plots (Supplementary Figure S8) drawn using fluorescence titration data suggest WP6 and the three guests in a 1:1 host–guest complex of the aqueous solution, respectively. Based on the nonlinear curve-fitting approach (Supplementary Figures S10, S11), the measured association constants (*K*_a_) were (2.51 ± 0.24) × 10^4^ M^−1^, (9.30 ± 0.04) × 10^3^ M^−1^, and (9.14 ± 0.08) × 10^3^ M^−1^ for caffeine, theophylline, and theobromine, respectively. The *K*_a_ value for the binding of caffeine is approximately an order of magnitude greater than the binding of theophylline and theobromine.

### Fluorescent Indicator Displacement

Next, the indicator displacement process was used to measure the nonfluorescent host–indicator complex to detect caffeine. Figure 3B displays the typical process of displacement titration. When caffeine was gradually added into a mixed PBS solution with ST and WP6, fluorescence intensity quenching was apparent. The result proved that the added caffeine was able to rival with ST to push the indicator from the WP6 cavity. The following optical changes could be attributed to the formation of the caffeine⊂WP6 complex, which exhibited higher stability compared with ST⊂WP6. Furthermore, the ‘turn-off’ fluorescence changes resulting from caffeine addition could be observed by naked eyes by using a simple UV lamp (Figure 3B ). The results indicate that the applicability of the ST⊂WP6 complex to an F-IDA for sensing anticancer drug caffeine.

**FIGURE 4:**
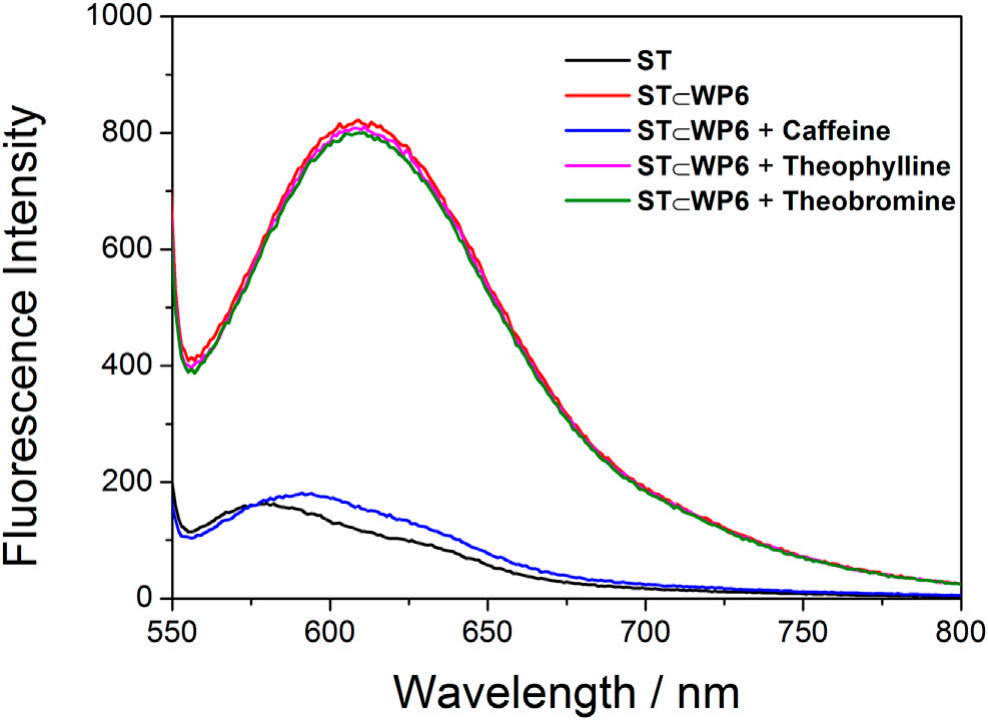
Fluorescence spectra for ST (0.02 mM) + WP6 (0.35 mM) with caffeine (2 mM), theophylline (2 mM), and theobromine (2 mM), respectively.

The ST⊂WP6 system as a caffeine sensor exhibited higher selectivity than theophylline and theobromine. Theophylline and theobromine at the same concentration (2 mM) were added to the solution of the ST⊂WP6 complex, respectively. Figure 4 displays changes of the fluorescence ratio I/I0 in the ST⊂WP6 complex on the addition of theophylline and theobromine, respectively. Negligible fluorescence changes were observed following the addition of theophylline and theobromine. Under the used conditions, theophylline and theobromine induced limited interference in the caffeine selective responses, suggesting the prominent selectivity of the method for caffeine. This selectivity can be attributed to the difference in binding constants between the host WP6 and the guests.

## Conclusion

In conclusion, a new host–indicator composed of an electron-deficient dye ST and anionic water-soluble pillar[6] arene WP6 was developed. After the ST⊂WP6 complex was formed, the twisted intramolecular charge-transfer-induced fluorescence enhancement and solution colour changes were apparent. Furthermore, this supramolecular system was successfully applied as a fluorescent indicator displacement assay to detect caffeine. Large signal modulation and a selective response towards caffeine against theophylline and theobromine were observed.

## Data Availability

The original contributions presented in the study are included in the article/Supplementary Material, further inquiries can be directed to the corresponding author.
