# Supplementary material for: Carboxylato-Pillar[6]arene-Based Fluorescent Indicator Displacement Assays for Caffeine Sensing
Source: Front Chem. 2021 Dec 21;9:816069. doi: 10.3389/fchem.2021.816069 (PMC8725816; doi:10.3389/fchem.2021.816069)
Supplement: Supplementary file 1 [file DataSheet1.DOCX]

Supplementary Material

1. *^1^H NMR spectra of substrates (****caffeine****,* ***theophylline****,* ***theobromine****) in the absence and presence of* ***WP6***


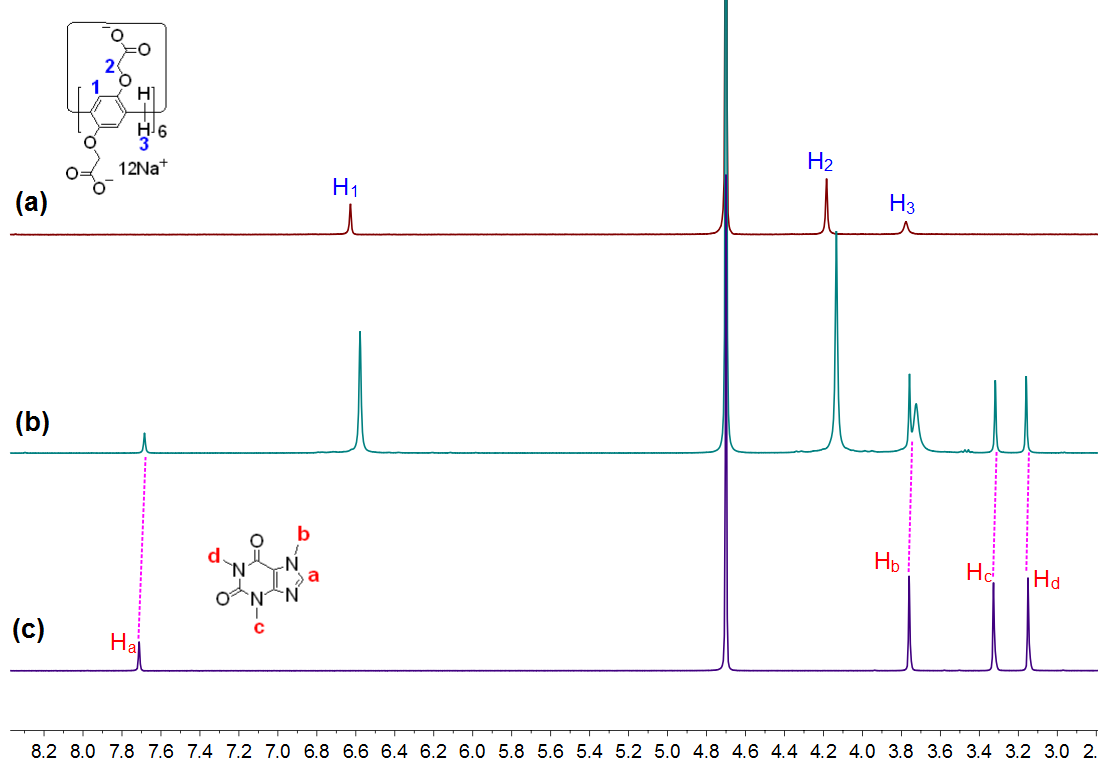


**Supplementary Figure 1.** ^1^H NMR spectra (400 MHz, D_2_O, 293 K) of (a) 10.0 mM **WP6**, (b) 10.0 mM **WP6** + 10.0 mM **caffeine**, and (c) 10.0 mM **caffeine**.


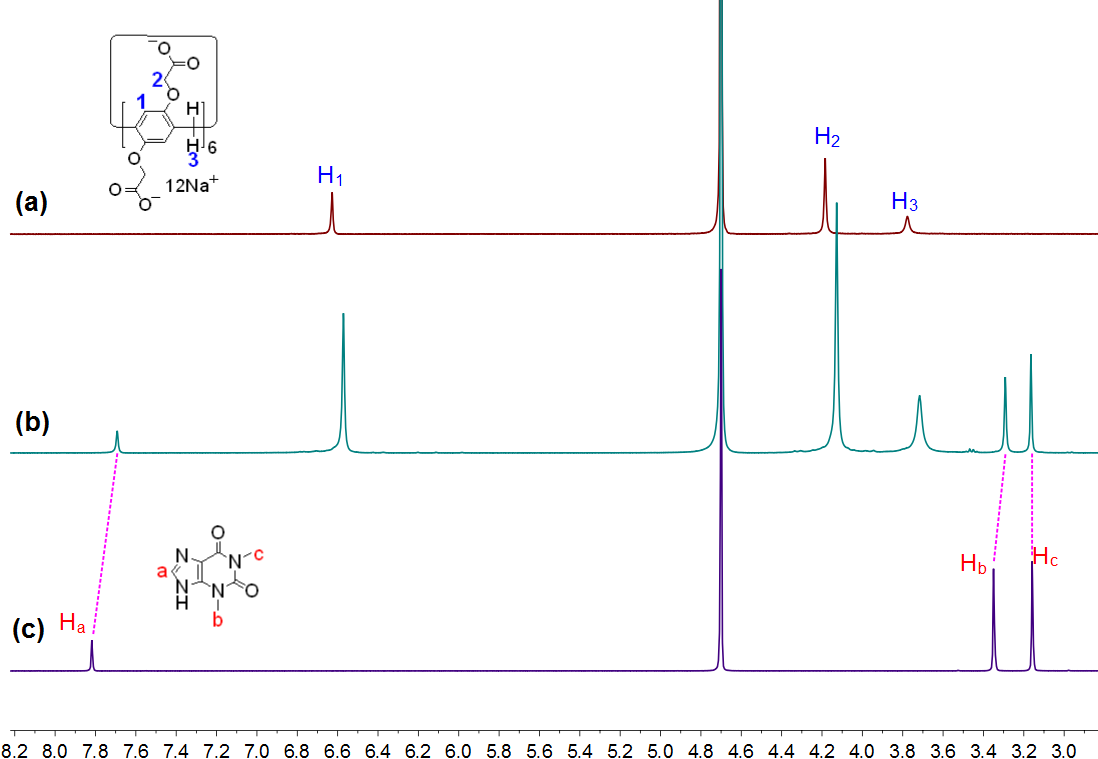


**Supplementary Figure 2.** ^1^H NMR spectra (400 MHz, D_2_O, 293 K) of (a) 10.0 mM **WP6**, (b) 10.0 mM **WP6** + 10.0 mM **theophylline**, and (c) 10.0 mM **theophylline**.


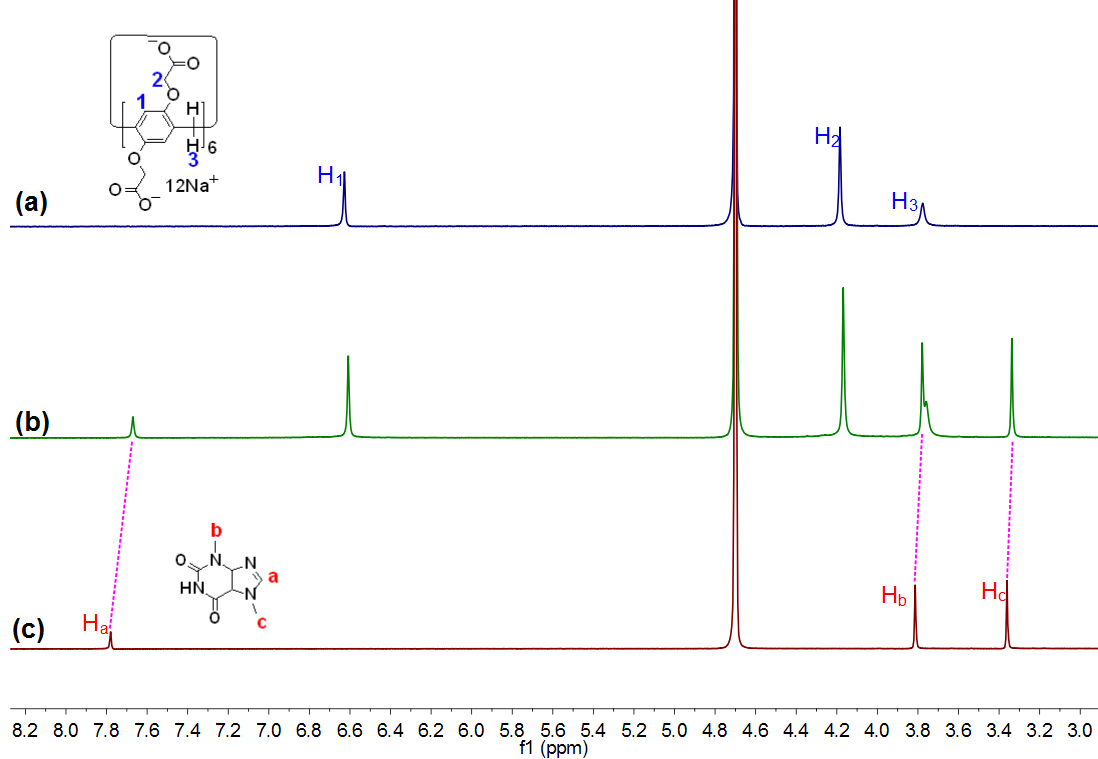


**Supplementary Figure 3.** ^1^H NMR spectra (400 MHz, D_2_O, 293 K) of (a) 10.0 mM **WP6**, (b) 10.0 mM **WP6** + 10.0 mM **theobromine**, and (c) 10.0 mM **theobromine**.

1. *2D NOESY spectra of* ***WP6****⊃* ***caffeine*** */* ***theophylline*** */* ***theobromine*** *complexes*


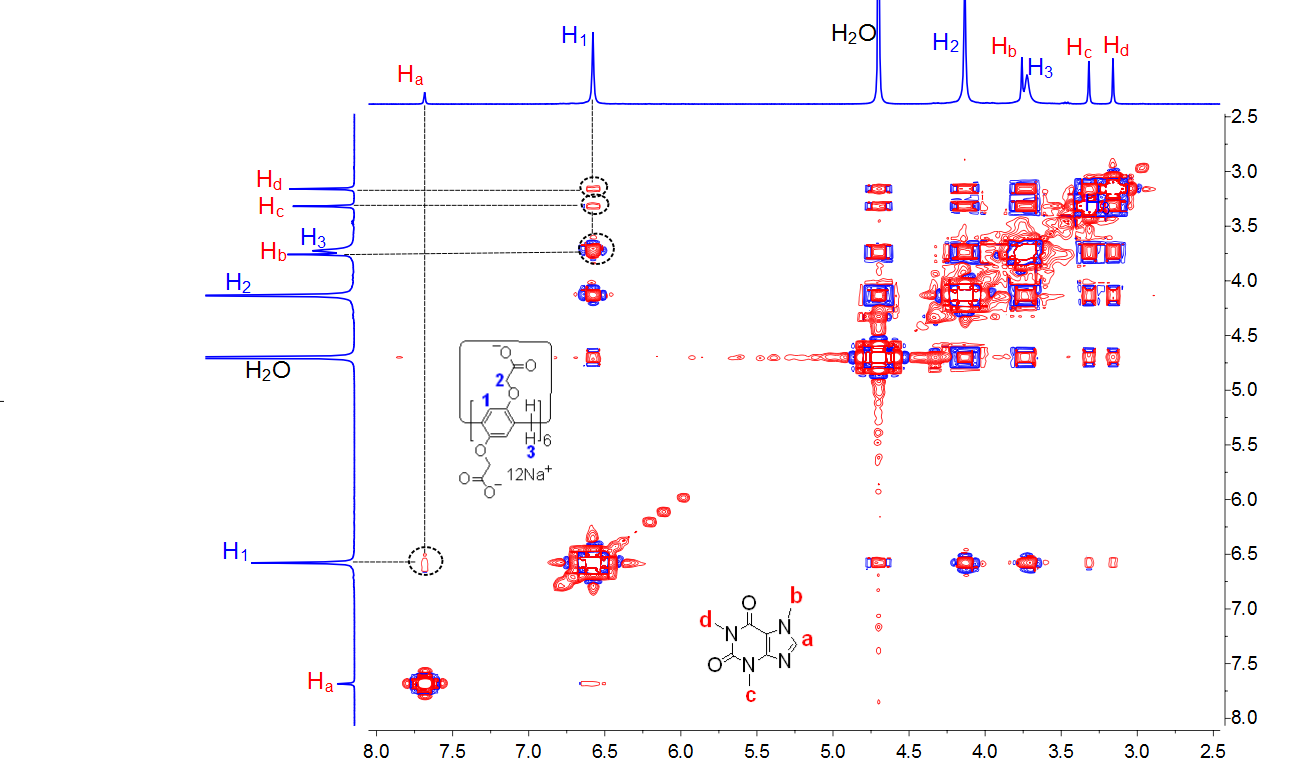


**Supplementary Figure 4.** 2D NOESY NMR spectrum of **WP6**⊃**caffeine** (400 MHz, D_2_O, 298 K, mixing time = 300 ms), [**WP6**] = 10.0 mM, [**caffeine**] = 10.0 mM.


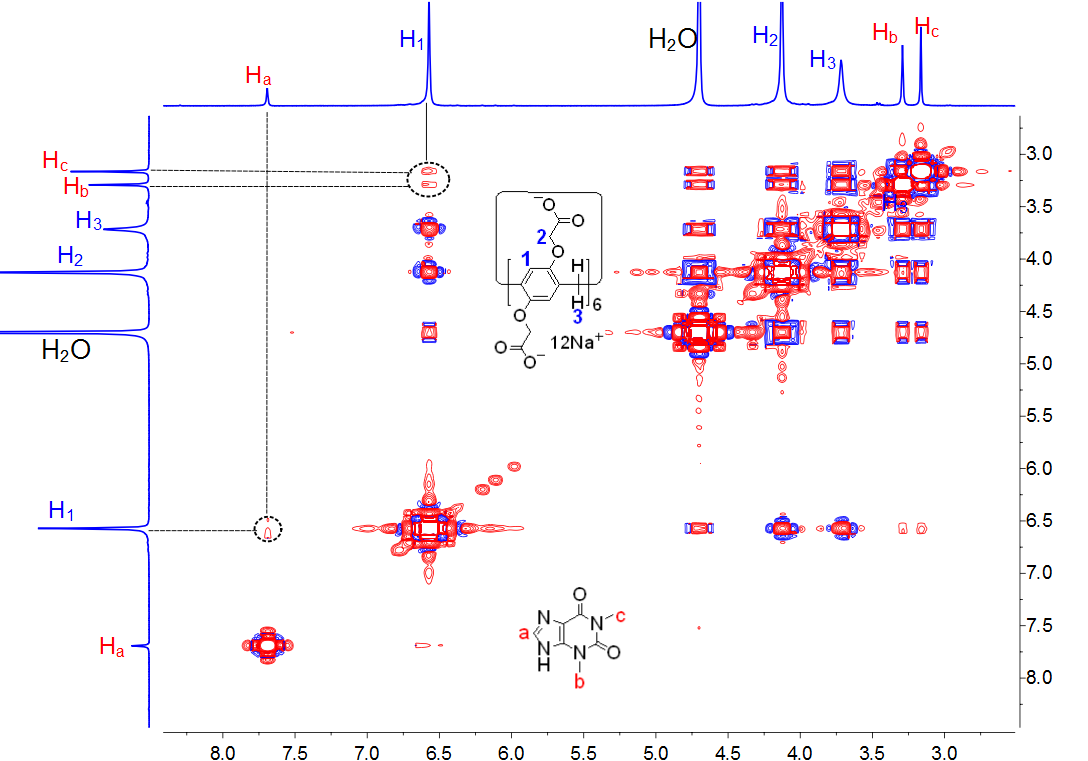


**Supplementary Figure 5.** 2D NOESY NMR spectrum of **WP6**⊃**theophylline** (400 MHz, D_2_O, 298 K, mixing time = 300 ms), [**WP6**] = 10.0 mM, [**theophylline**] = 10.0 mM.


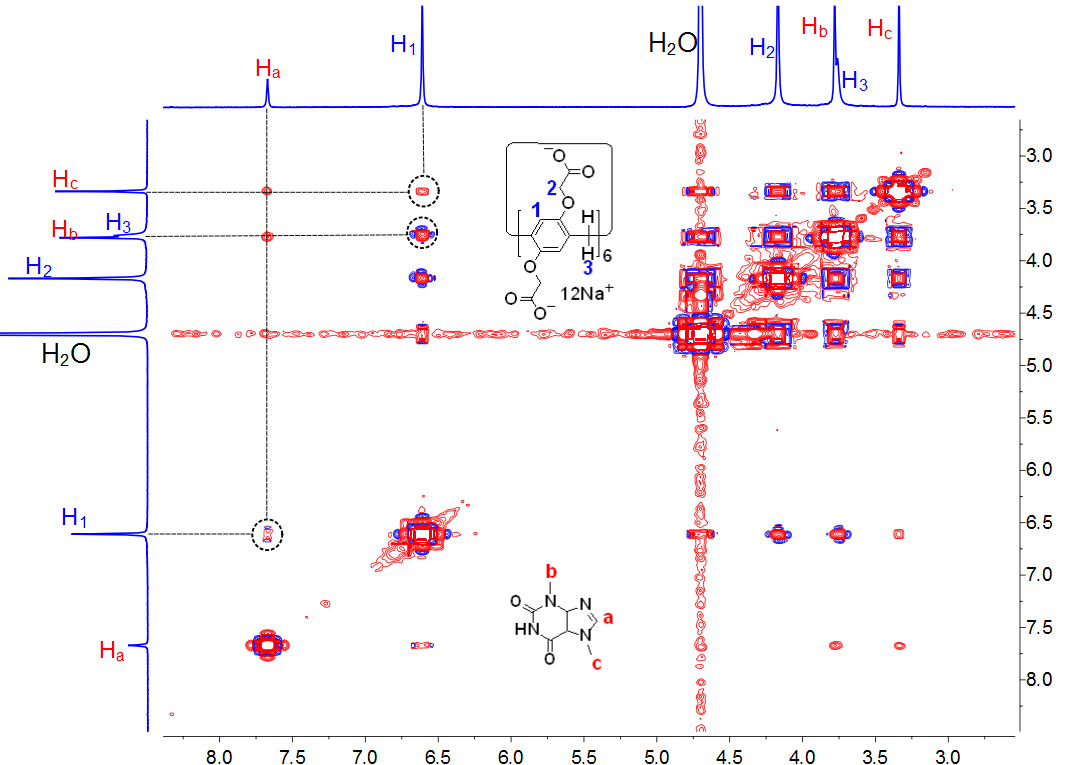


**Supplementary Figure 6.** 2D NOESY NMR spectrum of **WP6**⊃**theobromine** (400 MHz, D_2_O, 298 K, mixing time = 300 ms), [**WP6**] = 10.0 mM, [**theobromine**] = 10.0 mM.

*3. Determination of the association constants between substrates (****caffeine****,* ***theophylline****,* ***theobromine, and ST****) and* ***WP6***

*3.1 Job plot for* ***WP6****⊃* ***caffeine*** */* ***theophylline*** */* ***theobromine***










**Supplementary Figure 7.** (a-c) Fluorescence spectra of the mixture of **WP6** and substrates (**caffeine, theophylline, theobromine**) in water at different molar ratios while [**WP6**] + [substrates] = 1.0 × 10^−5^ M, respectively.










**Supplementary Figure 8.** (a-c) Job plot showing the 1:1 stoichiometry of the complex between **WP6** and substrates (**caffeine, theophylline, theobromine**) by plotting the difference in fluorescent emission intensity at *λ*_emission_ = 326 nm (*λ*_excitation_ = 290 nm) against the mole fraction of substrates at an invariant total concentration of 0.01 mM in aqueous solution, respectively.

*3.2 Association constants of* ***WP6****⊃* ***caffeine/theophylline/theobromine/ST***

To determine the association constants for the complexation between **WP6** and substrates (**caffeine, theophylline, theobromine,** and **ST**), fluorescence titration experiments were carried out in solutions which had a constant concentration of **WP6** (1.0 × 10^–5^ M) and varying concentrations of substrates. By a non-linear curve-fitting method, the association constants (*K*_a_) of ***WP6****⊃* ***caffeine/theophylline/theobromine/ST*** were estimated.

The non-linear curve-fittings were based on the equation:

*ΔF* = (*ΔF*_∞_/[H]_0_) (0.5[G]_0_ + 0.5([H]_0_+1/*K*_a_)−(0.5 ([G]_0_^2^+(2[G]_0_(1/*K*_a_ − [H]_0_)) + (1/*K*_a_ + [H]_0_)^2^) ^0.5^)) (eq. 1)

Where *ΔF* is the fluorescence intensity changes at 326 nm at [H]_0_, *ΔF_∞_* is the fluorescence intensity changes at 326 nm when **WP6** is completely complexed, [G]_0_ is the initial concentration of substrates (**caffeine, theophylline, theobromine, and ST**), and [H]_0_ is the fixed initial concentration of **WP6**.^S1^












**Supplementary Figure 9.** (a-d) Fluorescence spectra of **WP6** (1.0 × 10^–5^ M) upon addition of substrates (**caffeine, theophylline, theobromine, and ST**) (0-5.43 × 10^–4^ M) in aqueous solution (excited at 290 nm) at room temperature, respectively.










**Supplementary Figure 10.** The fluorescence intensity changes of **WP6** upon addition of substrates (**caffeine, theophylline, theobromine**), respectively. The red solid line was obtained from the non-linear curve-fitting using eq. 1.







**Supplementary Figure 11.** (a) Mole ratio plot for **WP6** and **ST**, indicating a 1:1 stoichiometry. (b) The fluorescence intensity changes of **WP6** upon addition of **ST**. The red solid line was obtained from the non-linear curve-fitting using eq. 1.

**Supplementary Table 1*.*** The association constants between substrates (**caffeine, theophylline, theobromine, and ST**) and **WP6** by fluorescence titration experiments.

| Host (guest) | *K*_a_ (L⋅mol^-1^) | Host (guest) | *K*_a_ (L⋅mol^-1^) |
| --- | --- | --- | --- |
| **WP6** (**caffeine**) | (2.51 ± 0.24) × 10^4^ | **WP6** (**theobromine**) | (9.14 ± 0.08) × 10^3^ |
| **WP6** (**theophylline**) | (9.30 ± 0.04) × 10^3^ | **WP6** (**ST**) | (1.50 ± 0.06) × 10^4^ |

*References:*

- S1. (a) K. A. Connors, Binding Constants, Wiley: New York, 1987. (b) P. S. Corbin, Ph.D. *Dissertation*, University of Illinois at Urbana-Champaign, Urbana, IL, 1999. (c) P. R. Ashton, R. Ballardini, V. Balzani, M. Belohradsky, M. T. Gandolfi, D. Philp, L. Prodi, F. M. Raymo, M. V. Reddington, N. Spencer, J. F. Stoddart, M. Venturi, D. J. Williams, *J. Am. Chem. Soc.* 118 (1996) 4931−4951. (d) J. Zhang, F. Huang, N. Li, H. Wang, H. W. Gibson, P. Gantzel, A. L. Rheingold, *J. Org. Chem.* 72 (2007) 8935−8938.
